# *Brucella melitensis* bacteraemia with lymphoma-like generalised lymphadenopathy and probable secondary haemophagocytic lymphohistiocytosis under biopsy constraints: a case report

**DOI:** 10.3389/fimmu.2026.1975515

**Published:** 2026-10-21

**Authors:** Xiaoqi Liu, Anning Song, Mingxia Li, Juning Ma, Qinglin Wang, Jing Yang

**Affiliations:** 1 First Clinical Medical College of Shandong University of Traditional Chinese Medicine, Jinan, Shandong, China; 2 ZiBo 148 Hospital, China RongTong Medical Healthcare Group Co. Ltd., Zibo, Shandong, China; 3 Department of Hematology, Weifang Traditional Chinese Medicine Hospital, Weifang, Shandong, China

**Keywords:** *Brucella melitensis*, case report, lymphadenopathy, lymphoma mimic, probable secondary haemophagocytic lymphohistiocytosis

## Abstract

**Background:**

Brucellosis can resemble lymphoma when lymphadenopathy accompanies cytopenias and systemic inflammation. Associated secondary haemophagocytic lymphohistiocytosis (HLH) adds to the diagnostic difficulty.

**Case presentation:**

A 68-year-old man presented with anorexia, generalised lymphadenopathy, splenomegaly, cytopenias, and hepatic and renal dysfunction. Fever developed on the first hospital evening and reached 41 °C on day 2. Empirical antibiotics and corticosteroids were started before microbiological confirmation. Severe thrombocytopenia complicated biopsy planning, and the patient declined tissue sampling because of financial hardship and concern about physical harm. Blood cultures collected on day 2 yielded *Brucella melitensis*, reported on day 6. Doxycycline and rifampicin were started, and methylprednisolone was continued. Five HLH-2004 criteria and a retrospective HScore of 234 supported probable secondary HLH. Fever and some laboratory abnormalities improved during empirical treatment, but cytopenias persisted at discharge on day 10. Follow-up blood tests approximately 1 month after discharge showed recovery of white-cell, neutrophil and platelet counts to reference ranges, although anaemia persisted. At approximately 15 months, the patient reported feeling well but declined further laboratory testing and imaging.

**Conclusion:**

Blood cultures identified *B. melitensis* infection in a patient with lymphoma-like findings and probable secondary HLH. Microbiological identification and serial assessment guided treatment when tissue sampling was declined.

## 1 Introduction

Brucellosis is a zoonotic infection caused by Brucella species. *B. melitensis*, acquired mainly from sheep and goats, is the most common and most virulent species in humans (1, 2). Infection follows contact with infected animals or their birth products, ingestion of unpasteurised dairy products, or inhalation of contaminated aerosols, and the disease remains endemic in the Mediterranean basin, the Middle East, Asia, Africa, and Latin America (1, 2). Brucellosis has no single defining clinical pattern. Fever, sweating, fatigue, anorexia, weight loss, and arthralgia are the most frequent complaints, and focal disease may involve the bones and joints, genitourinary tract, liver, heart, or nervous system (3, 4). Laboratory abnormalities are equally nonspecific and include anaemia, leucopenia, thrombocytopenia or pancytopenia, raised C-reactive protein, and abnormal liver enzymes (4). Diagnosis depends on culture, serology, or nucleic acid amplification; blood culture is the reference standard but may need several days of incubation (5).

Brucella is a facultative intracellular pathogen that survives within mononuclear phagocytes and therefore preferentially involves the reticuloendothelial system (2). Imaging may show hepatomegaly, splenomegaly, or lymphadenopathy (4). When these findings accompany fever and cytopenias, the initial impression can shift towards lymphoma or tuberculosis before brucellosis is considered (6).

Secondary haemophagocytic lymphohistiocytosis (HLH) is a hyperinflammatory syndrome caused by uncontrolled activation of cytotoxic lymphocytes and macrophages, with excessive cytokine release and tissue damage (7). In adults it is usually triggered by infections, malignancies, or autoimmune and autoinflammatory diseases (7). Viral infections, particularly Epstein–Barr virus, are the most frequently reported infectious triggers, but bacterial, mycobacterial, parasitic, and fungal infections, including intracellular bacteria such as Brucella, have also been described (8). Lymphoma is the leading malignant trigger, and in autoimmune disease the syndrome presents as macrophage activation syndrome, most often in adult-onset Still’s disease and systemic lupus erythematosus (9). Fever, splenomegaly, cytopenias, hyperferritinaemia, hypertriglyceridaemia, and hypofibrinogenaemia are characteristic, and the majority of these features also occur in severe infection and in lymphoma. The HLH-2004 criteria and the HScore support recognition of the syndrome but do not identify its cause (10, 11).

The overlap between brucellosis and lymphoma is therefore bidirectional: brucellosis can resemble lymphoma through its nodal and haematological features, and lymphoma is itself a common cause of secondary HLH. Lymphoma-like presentations of brucellosis and Brucella-associated HLH have each been reported, and in several HLH cases the diagnosis was supported by bone-marrow haemophagocytosis (12–16). The present case combines both problems in one patient. Culture-confirmed *B. melitensis* bacteraemia was accompanied by generalised lymphadenopathy, a peripancreatic soft-tissue lesion, and probable secondary HLH, whilst lymph-node biopsy and bone-marrow examination were not performed because the patient declined them and severe thrombocytopenia increased the procedural risk. The exposure setting emerged only from seasonal work outside the patient’s recorded occupation. We describe the stepwise diagnostic and treatment decisions, what the non-invasive assessment could and could not exclude, and the subsequent clinical course and follow-up.

## 2 Case presentation

### 2.1 Presenting complaint, baseline history, and initial differential diagnosis

A 68-year-old man was admitted to the haematology department with more than two weeks of anorexia and poor oral intake. A local clinic had found a glucose concentration of 7.75 mmol/L and sinus tachycardia and prescribed drugs to lower blood glucose, blood pressure, and heart rate; the drug names and doses were not recorded. Further assessment at local facilities showed leucopenia, severe thrombocytopenia, raised aminotransferases, lactate dehydrogenase (LDH) of 1093 U/L, and creatinine of 201 µmol/L. Contrast-enhanced computed tomography (CT) on 31 May 2025 showed enlarged lymph nodes in the bilateral supraclavicular regions, mediastinum, and retroperitoneum, a soft-tissue lesion anterior-inferior to the pancreatic head, splenomegaly, and small-volume ascites (**Figure 1**). The radiological impression favoured a lymphoid neoplasm.

**Figure 1:**
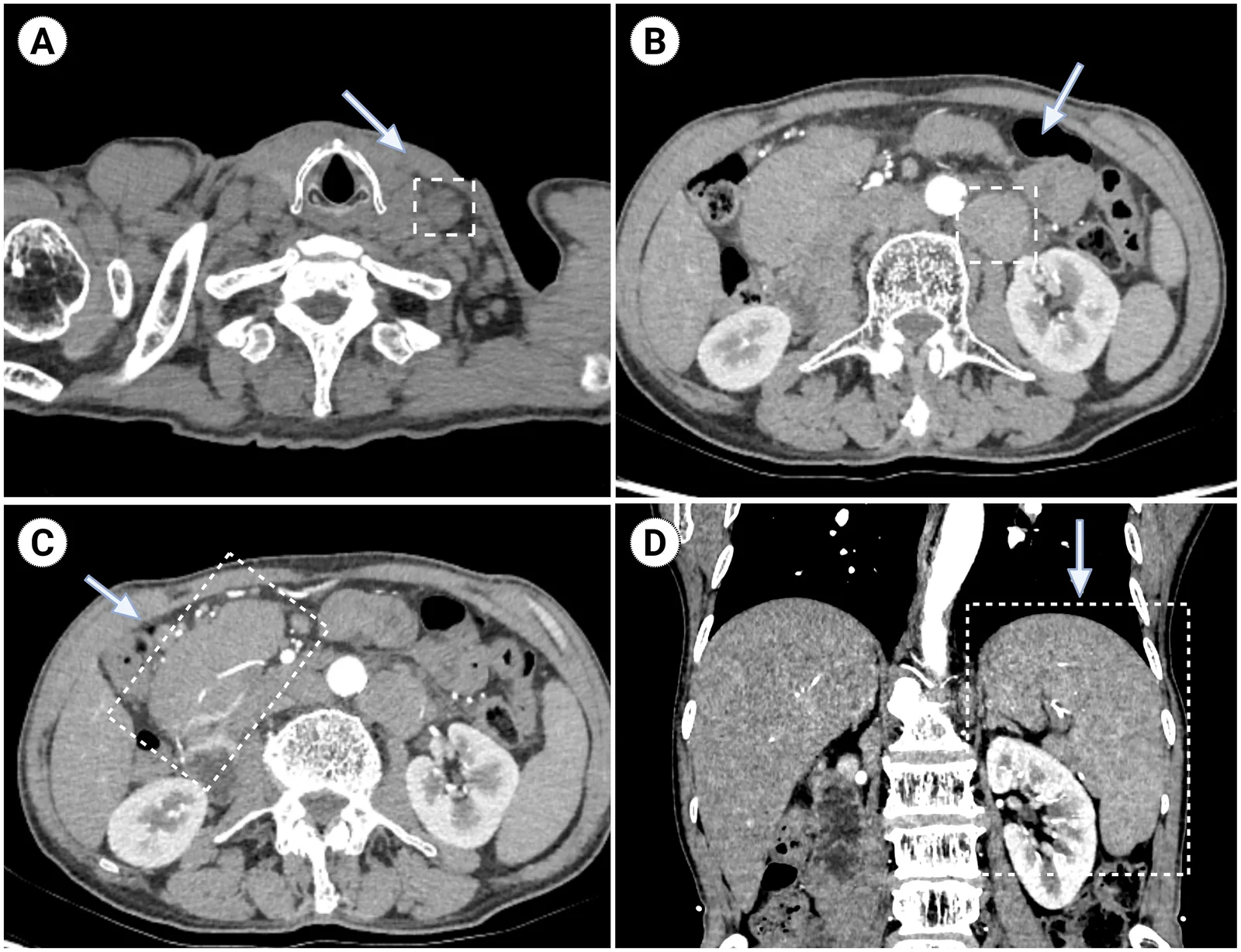
Lymphoma-like imaging findings in brucellosis. **(A)** Enlarged left supraclavicular lymph nodes. **(B)** Retroperitoneal lymphadenopathy. **(C)** A soft-tissue lesion or nodal conglomerate anterior-inferior to the pancreatic head. **(D)** Splenomegaly. Dashed outlines mark the regions described. The pre-admission CT findings raised concern for a lymphoid neoplasm.

Subsequent local-hospital records and clarification confirmed a history of type 2 diabetes treated with unspecified medication, although this history had not been accurately captured at the initial admission. At the initial admission, the patient reported no established hypertension, hepatitis, tuberculosis, cardiac disease or cerebrovascular disease. No relevant hereditary family history was reported, and no pre-admission corticosteroid or other immunosuppressive treatment was documented. He had smoked approximately 20 cigarettes daily for more than 40 years and drank alcohol daily until about one week before admission. His recorded occupation was farming (**Supplementary Table S1**).

On admission he was afebrile (36.5 °C), thin, and in no acute distress, and reported fatigue, throat discomfort, and dry mouth without recent weight loss or night sweats. The abdomen was soft and non-tender. No peripheral lymph node was palpable, and neither liver nor spleen was palpable below the costal margin. The first hospital blood sample showed leucopenia, mild anaemia, severe thrombocytopenia, raised C-reactive protein, and hepatic and renal abnormalities (**Table 1**). Nutritional assessment on day 3 identified severe malnutrition (**Table 2**; **Supplementary Table S1**).

**Table 1:** Presentation and first hospital blood sample.

Assessment	Finding
Presentation	68-year-old man; anorexia and poor intake for >2 weeks; admitted 1 June 2025.
Examination	Temperature 36.5 °C; thin; abdomen soft and non-tender; no palpable peripheral lymphadenopathy; liver and spleen not palpable.
CT, 31 May	Supraclavicular, mediastinal, and retroperitoneal lymphadenopathy; soft-tissue lesion near pancreatic head; splenomegaly; small-volume ascites.
Blood count	WBC 2.21 × 10^9^/L; ANC 1.06 × 10^9^/L; RBC 3.21 × 10¹²/L; haemoglobin 112 g/L; platelets 28 × 10^9^/L.
Inflammation and biochemistry	hs-CRP 48.68 mg/L; ALT 72.6 U/L; AST 206.9 U/L; albumin 26.6 g/L; total bilirubin 24.9 µmol/L; creatinine 135 µmol/L; β2-microglobulin 7.73 mg/L.

ALT, alanine aminotransferase; ANC, absolute neutrophil count; AST, aspartate aminotransferase; CT, computed tomography; hs-CRP, high-sensitivity C-reactive protein; RBC, red blood cells; WBC, white blood cells.

**Table 2:** Clinical timeline and rationale for investigations.

Date/hospital day	Clinical problem, investigations, and rationale	Treatment or decision
Before admission	Anorexia, cytopenias, and hepatic/renal abnormalities; CT suggested lymphoma.	Referral to haematology.
1	Afebrile initially; baseline blood sample. Fever developed that evening.	Piperacillin–tazobactam for suspected infection; dexamethasone after fever developed.
2	Fever to 41 °C and drowsiness. Paired blood cultures. Procalcitonin and cytokine panel to characterise the inflammatory response. D-dimer 21.23 mg/L.	Methylprednisolone before procalcitonin/cytokine results. Imipenem stopped after rash. One dose of low-molecular-weight heparin. SFTS testing recommended.
3	No fever at the medical ward round; mobile, non-tender left infraclavicular node. Autoimmune profile and viral IgM to screen alternative triggers; fungal markers because of fever on broad-spectrum antibiotics and corticosteroids (all negative). Nutrition review.	Levofloxacin added. SFTS order cancelled after refusal at collection.
4	Platelets 9 × 10^9^/L reported from a day 3 specimen. Surgical review: left cervical node about 1.5 × 1.0 cm, poor mobility, indistinct margins.	Platelet support then node biopsy recommended; biopsy declined because of cost and fear of harm. Meropenem added; methylprednisolone reduced to 20 mg.
5	HFRS IgM negative (fever, thrombocytopenia, renal dysfunction). Pretransfusion platelets later reported as 30 × 10^9^/L.	Methylprednisolone back to 40 mg. One therapeutic unit of platelets transfused.
6	*B. melitensis* reported. Persistent cytopenias prompted ferritin, triglyceride, and LDH testing for suspected HLH.	Levofloxacin and meropenem stopped that morning. Transfer to infectious diseases; doxycycline and rifampicin that afternoon. Methylprednisolone continued; marrow examination declined.
7	Ferritin >2000 µg/L, fibrinogen 1.49 g/L, triglycerides 2.74 mmol/L: probable HLH. Repeat critical D-dimer 16.21 mg/L prompted venous Doppler to exclude thrombosis before considering further anticoagulation.	Antibacterial and corticosteroid treatment continued. Anticoagulation deferred.
8	Vascular surgical review of thrombosis and bleeding risk.	Advised against anticoagulation because of thrombocytopenia.
10	Inflammatory indices improved; cytopenias persisted.	Discharge against medical advice for financial reasons; continued local care and doxycycline–rifampicin advised. Methylprednisolone was continued after discharge.
From 11 June 2025	Follow-up examinations and blood tests at a nearby hospital after returning home; blood counts remained low during June.	Received methylprednisolone 40 mg IV daily, ceftriaxone 3 g IV daily, and amikacin 0.4 g IV daily.
1 and 19 July 2025	WBC, ANC, and platelets within local reference ranges on both dates. Haemoglobin 102 and 111 g/L, respectively; residual anaemia. ALT and AST normal.	Objective laboratory follow-up (**Supplementary Table S4**).
18 September 2026 (~15 months)	Patient reported feeling well and no longer noticing neck swelling.	Further blood tests and imaging declined because of cost, travel, and perceived recovery.

Multiregional lymphadenopathy, a peripancreatic lesion, splenomegaly, cytopenias, and raised LDH made lymphoma the leading admission diagnosis, and gastrointestinal malignancy was also recorded in the admission differential. Because the presentation also included systemic inflammation and hepatic and renal dysfunction, infectious causes of lymphadenopathy and cytopenia, including disseminated tuberculosis and viral infection, and autoimmune disease were assessed as the course evolved (**Table 3**).

**Table 3:** Differential diagnosis and extent of assessment.

Condition	Reason considered	Investigations and findings
Brucellosis	Fever, cytopenias, splenomegaly, and lymphadenopathy; exposure setting identified later.	*B. melitensis* in both aerobic bottles (69–73 h). Serology and nucleic acid testing not performed.
Lymphoma	Multiregional lymphadenopathy, peripancreatic lesion, splenomegaly, cytopenias, raised LDH, and β2-microglobulin; CT favoured a lymphoid neoplasm.	Node biopsy recommended on day 4 and declined; marrow examination declined on day 6. No repeat imaging.
Gastrointestinal or other malignancy	Anorexia; peripancreatic soft-tissue lesion.	No endoscopy or tissue sampling.
Disseminated tuberculosis	Lymphadenopathy, fever, and cytopenias.	No previous tuberculosis; chest CT without cavitation or miliary change (**Supplementary Figure S1**). No tuberculosis-specific test.
Autoimmune disease	Cytopenias and systemic inflammation.	ANA, anti-dsDNA, anti-Sm, anti-SSA, and anti-SSB within reference ranges.
Viral infection	Fever, cytopenias, and hepatic and renal dysfunction.	EBV VCA IgM, CMV IgM, and HFRS IgM negative. SFTS testing refused; HIV not tested.
Invasive fungal infection	Fever during broad-spectrum antibiotics and corticosteroids.	1,3-β-D-glucan and galactomannan negative.

ANA, antinuclear antibody; CMV, cytomegalovirus; dsDNA, double-stranded DNA; EBV VCA, Epstein–Barr virus viral capsid antigen; HFRS, haemorrhagic fever with renal syndrome; LDH, lactate dehydrogenase; SFTS, severe fever with thrombocytopenia syndrome.

### 2.2 Deterioration, investigations, and empirical treatment (days 1–5)

Intravenous piperacillin–tazobactam 4.5 g twice daily was started on day 1 for suspected infection. Fever developed that evening, and dexamethasone 5 mg was administered intravenously. On day 2 the temperature reached 41 °C and the patient became drowsy. With cytopenias and hepatic and renal dysfunction already present and the cause still undefined, methylprednisolone 40 mg intravenously was prescribed as an empirical anti-inflammatory adjunct to antibiotics and fluids. Imipenem–cilastatin was substituted on day 2 but stopped that morning after a rash, which was treated with promethazine, and piperacillin–tazobactam was resumed.

Paired blood-culture sets were drawn from both arms on day 2, after antibiotics and the first corticosteroid dose had been administered. Procalcitonin and a Th1/Th2 cytokine panel were ordered during the deterioration to characterise the inflammatory response, because high fever with cytopenias may reflect bacterial sepsis or a cytokine-driven hyperinflammatory state. Procalcitonin was 0.966 ng/mL; interleukin-6 (IL-6), IL-10, and interferon-γ were 553.65, 56.41, and 20.18 pg/mL, respectively. These results became available after methylprednisolone had been prescribed and did not inform that decision. D-dimer was 21.23 mg/L and fibrinogen 1.62 g/L; the critical D-dimer prompted one subcutaneous dose of low-molecular-weight heparin 5,000 IU. An infectious diseases consultation recommended nucleic acid testing for severe fever with thrombocytopenia syndrome (SFTS), a tick-borne viral infection that also causes fever, leucopenia, and thrombocytopenia. The order was later cancelled after the patient refused specimen collection.

At the medical ward round on day 3 no fever was recorded, and a mobile, non-tender left infraclavicular node was palpable. Alternative causes of the illness were screened in parallel. The autoimmune antibody profile, obtained to look for systemic autoimmune disease as a cause of cytopenia and hyperinflammation, was within reference ranges, including antinuclear antibody, anti-double-stranded DNA, anti-Sm, anti-SSA, and anti-SSB. Epstein–Barr virus viral capsid antigen IgM and cytomegalovirus IgM were negative. Serum 1,3-β-D-glucan and Aspergillus galactomannan, measured because the patient was febrile whilst receiving broad-spectrum antibiotics and corticosteroids, were negative on day 3. Haemorrhagic fever with renal syndrome IgM, relevant to the combination of fever, thrombocytopenia, and renal dysfunction, was negative on day 5. Levofloxacin was added on day 3 and meropenem on day 4 during empirical treatment.

On day 4, the platelet nadir of 9 × 10^9^/L was reported from a day 3 specimen. Surgical review described a left cervical node of approximately 1.5 × 1.0 cm with poor mobility and indistinct margins. The surgeon recommended platelet support followed by excisional or ultrasound-guided node biopsy and explained the procedural risks. The patient declined consent because of cost and concern about physical harm. Methylprednisolone was reduced to 20 mg daily on day 4 and returned to 40 mg daily on day 5. One therapeutic unit of apheresis platelets was transfused on day 5 to reduce bleeding risk, without a recorded transfusion reaction. **Table 4** lists the principal treatment orders, and **Supplementary Table S1** gives supportive treatment and further details of treatment changes.

**Table 4:** Principal treatment orders.

Intervention	Dose/route/frequency	Timing and clinical context
Piperacillin–tazobactam	4.5 g IV twice daily	Days 1–2; resumed on day 2 after imipenem reaction and stopped on day 5. Empirical treatment of suspected infection.
Imipenem–cilastatin	0.5 g IV every 8 h prescribed	Day 2; stopped after rash. Promethazine 25 mg IM was administered.
Levofloxacin	100 mL IV daily; concentration not recorded	Days 3–6, empirical; stopped on the morning of day 6 before the species report.
Meropenem	1 g IV every 12 h	Days 4–6, empirical.
Doxycycline	100 mg orally every 12 h	From day 6 through discharge, after *B. melitensis* identification.
Rifampicin	600 mg orally daily	From day 6 through discharge, with doxycycline.
Dexamethasone	5 mg IV, one-off orders	Day 1 evening, day 2, and day 5; the day 5 order was around platelet transfusion.
Methylprednisolone	40 mg IV initial order; then daily orders	Day 2 initial and standing orders; 20 mg daily on day 4; 40 mg daily from day 5, continued after discharge.
Low-molecular-weight heparin	5,000 IU subcutaneously, once	Day 2 after critical D-dimer result; subsequent anticoagulation withheld because of bleeding risk.
Apheresis platelets	One therapeutic unit IV	Day 5, for severe thrombocytopenia; no transfusion reaction recorded.
Discharge antibacterial prescription	Doxycycline 100 mg every 12 h and rifampicin 600 mg daily, orally	A 42-day course was prescribed at discharge and subsequently completed.
Ceftriaxone	3 g IV daily	During continued treatment after returning home following discharge on 10 June 2025.
Amikacin	0.4 g IV daily	During continued treatment after returning home following discharge on 10 June 2025.

Doses and treatment days follow the medication orders. Levofloxacin is reported as the recorded infusion volume. IM, intramuscular; IV, intravenous. Supportive regimens are given in **Supplementary Table S1**. Hospital days refer to the initial admission.

### 2.3 Microbiological diagnosis, exposure history, and HLH assessment (days 6–8)

On day 6, both aerobic blood-culture bottles were reported to contain *B. melitensis*, with recorded incubation intervals of 69 h 29 min and 73 h 29 min. Levofloxacin had been stopped that morning before the species report, and meropenem was stopped later that morning. The patient was transferred to infectious diseases, and doxycycline 100 mg orally every 12 hours plus rifampicin 600 mg orally daily was started that afternoon; methylprednisolone 40 mg daily was continued. The family declined bone-marrow examination the same day. Brucella serology and nucleic acid testing were not performed.

On directed questioning after the culture result, the patient explained that outside the farming season he worked as a street cleaner on a route that passed several sheep farms. He reported no direct handling of livestock or birth products and no consumption of unpasteurised dairy products. This work provided a possible exposure setting, although the source of infection could not be confirmed.

Persistent cytopenias after identification of the organism prompted consideration of a haemophagocytic syndrome, and ferritin, triglycerides, and LDH were ordered. On day 7, ferritin exceeded the assay ceiling of 2000 µg/L, fibrinogen was 1.49 g/L, triglycerides 2.74 mmol/L, and LDH 397 U/L. Five HLH-2004 criteria were fulfilled during the course: fever, splenomegaly, cytopenias affecting at least two lineages, hypofibrinogenaemia, and hyperferritinaemia (10). The triglyceride/fibrinogen criterion was met by low fibrinogen; triglycerides were below the 3.0 mmol/L threshold. Marrow haemophagocytosis, natural killer cell activity, and soluble CD25 were unassessed rather than negative, because bone-marrow examination was declined and the latter two assays were unavailable at the treating centre.

The retrospective HScore was 234, using the recorded peak temperature, pre-admission imaging, and day 7 laboratory results. Ferritin above the assay ceiling received the minimum supported 35 points, unassessed marrow haemophagocytosis received 0 points, and known immunosuppression received 0 because none was documented. The score corresponds to a model probability of approximately 98%–99% and supports probable secondary HLH (**Figure 2**; **Supplementary Table S2**) (11).

**Figure 2:**
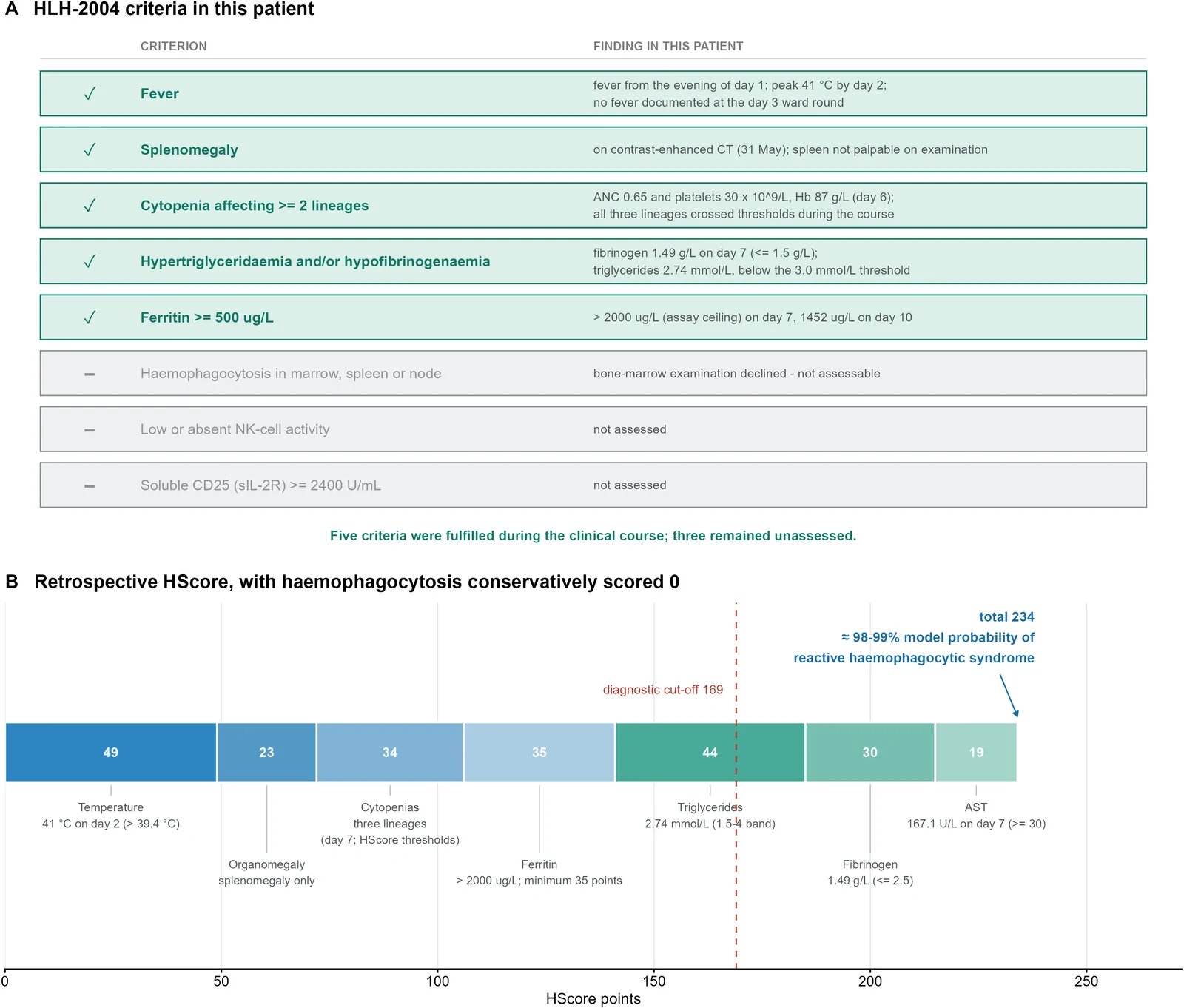
Assessment against HLH-2004 criteria and the HScore. **(A)** Five HLH-2004 criteria were fulfilled during the clinical course; three remained unassessed. Fibrinogen 1.49 g/L fulfilled the triglyceride/fibrinogen criterion; triglycerides 2.74 mmol/L were below the HLH-2004 threshold. **(B)** The retrospective HScore totalled 234 using peak temperature, pre-admission imaging, and day 7 laboratory results. Ferritin >2000 µg/L received the minimum supported 35 points. Marrow haemophagocytosis was unassessed and assigned 0; known immunosuppression received 0 because none was documented. The dashed line marks the published HScore cut-off of 169. The approximate 98%–99% model probability supports the syndrome assessment.

The day 7 and day 10 progress notes linked continued methylprednisolone to persistent cytopenias and the findings supporting suspected infection-associated HLH. Etoposide was not used. On day 7, a repeat critical D-dimer of 16.21 mg/L prompted lower-limb venous ultrasonography to look for deep-vein thrombosis before any decision on anticoagulation; no thrombosis was seen in the examined veins. Anticoagulation was deferred, and vascular surgery advised against it on day 8 because of thrombocytopenia and bleeding risk.

### 2.4 Clinical course, discharge, and follow-up

No fever was recorded at the day 3 medical ward round, and subsequent progress notes described stable temperatures. Creatinine of 79 µmol/L and IL-6 of 1.56 pg/mL, measured in day 5 specimens, were reported on day 6 before doxycycline and rifampicin were prescribed, so these changes occurred during empirical treatment; the two IL-6 measurements used different assays and specimen types. Between day 7 and day 10, ferritin fell from >2000 to 1452 µg/L, LDH from 397 to 301 U/L, and D-dimer from 16.21 to 5.34 mg/L, whilst fibrinogen rose to 2.58 g/L. Procalcitonin fell to 0.058 ng/mL and aspartate aminotransferase to 46.1 U/L by day 10 (**Figure 3**; **Supplementary Table S3**).

**Figure 3:**
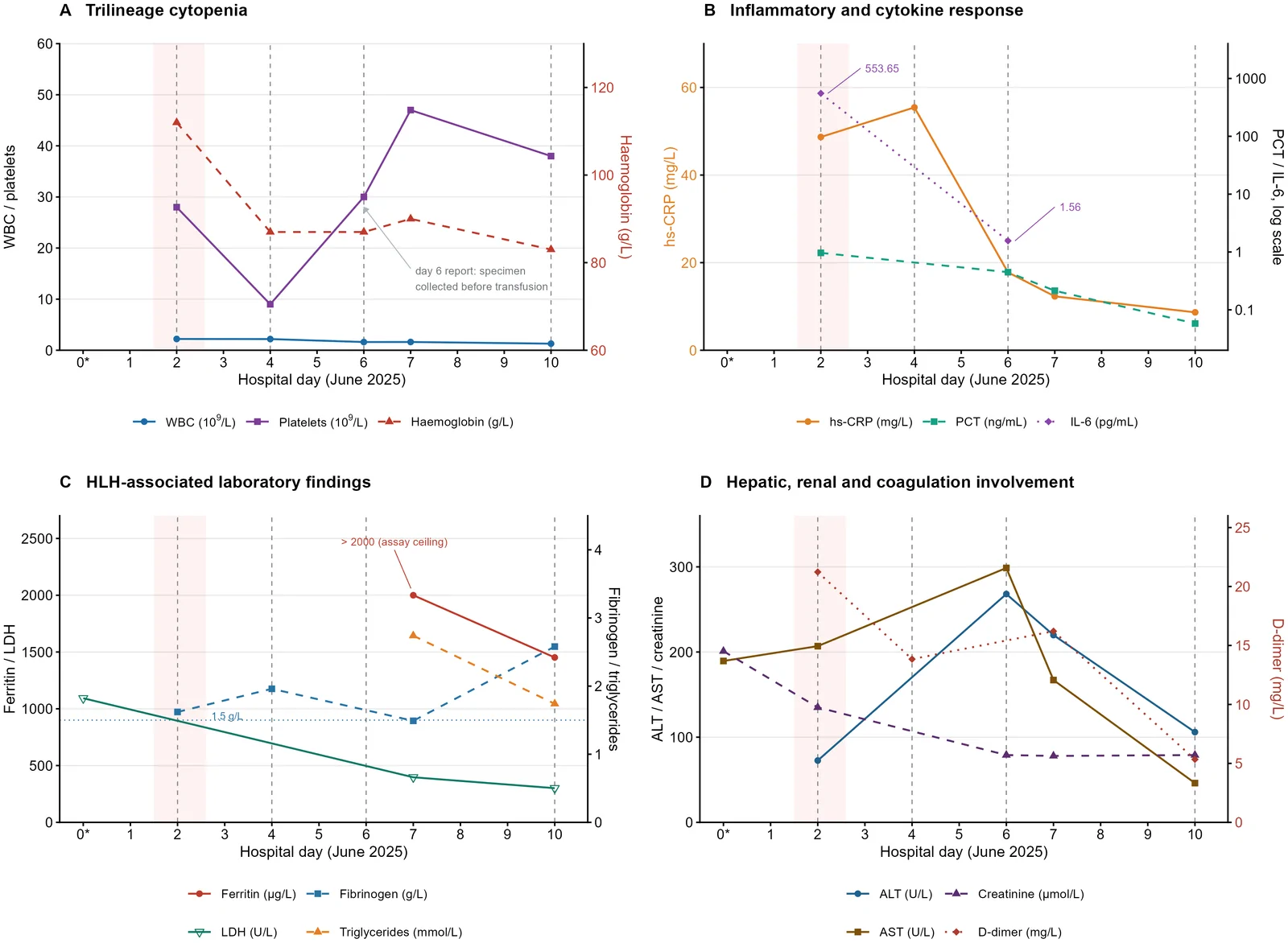
Dynamic laboratory changes during hospitalisation. **(A)** Blood counts. **(B)** Inflammatory markers. **(C)** Ferritin, lactate dehydrogenase, fibrinogen, and triglycerides. **(D)** Hepatic, renal, and D-dimer measurements. Points show results from one patient on report days; connecting lines guide the eye. Day 0 denotes referring-hospital results. Vertical dashed lines mark day 2 (clinical deterioration and blood-culture collection), day 4 (reported platelet nadir and surgical review), day 6 (species report and start of directed treatment), and day 10 (discharge). Pink shading marks the evening of day 1 through day 2, when fever was documented.

Cytopenias did not resolve. Platelets were 30 × 10^9^/L in the day 5 specimen collected before transfusion and 47 × 10^9^/L on day 7 after transfusion. On day 10, platelets were 38 × 10^9^/L, white cells 1.29 × 10^9^/L, and haemoglobin 83 g/L.

On day 10, the patient and family requested early discharge because of financial hardship and lack of medical insurance. The team explained that the blood counts remained abnormal and recommended continued care at a local hospital. The patient left against medical advice after the risks were discussed and acknowledged. His discharge instructions advised continuation of doxycycline 100 mg every 12 hours and rifampicin 600 mg daily and specified a 42-day course. Continued use and completion of the prescribed antibacterial course were subsequently confirmed, although the exact completion date was unavailable. Methylprednisolone 40 mg IV daily was continued after discharge.

After returning home, he underwent follow-up examinations and blood tests at a nearby hospital whilst continuing treatment (**Table 4**; **Supplementary Table S1**). Follow-up blood tests showed white-cell, neutrophil, and platelet counts within the local reference ranges on 1 and 19 July. By 19 July, haemoglobin had improved to 111 g/L but remained low; alanine and aspartate aminotransferases were both 17 U/L (**Supplementary Table S4**).

At telephone follow-up on 18 September 2026, approximately 15 months after discharge, the patient reported feeling well and no longer noticing neck swelling. He declined further blood tests and CT because of cost, travel difficulties, and his perceived recovery; this later outcome therefore remained patient-reported.

## 3 Discussion

### 3.1 Brucellosis as a lymphoma mimic

This case illustrates how *B. melitensis* bacteraemia complicated by probable secondary HLH can reproduce the clinical, laboratory, and imaging features that usually point to lymphoma. Multiregional lymphadenopathy, a peripancreatic soft-tissue lesion, splenomegaly, cytopenias, and raised LDH and β2-microglobulin all supported a lymphoid neoplasm at admission. These findings are nevertheless compatible with severe brucellosis, which involves the liver, spleen, lymph nodes, and bone marrow through persistence of the organism in mononuclear phagocytes (2, 4). The raised β2-microglobulin and LDH were also non-specific in the setting of systemic inflammation, hepatic injury, and renal dysfunction, and should not be interpreted as evidence of tumour burden in isolation.

Similar diagnostic pitfalls have been described. Massoud and colleagues reported a 52-year-old man with high fever, night sweats, and abdominal pain whose CT showed mesenteric and retroperitoneal lymphadenopathy suspicious for lymphoma; *B. melitensis* was confirmed by blood culture and serology, and the nodes resolved after treatment (12). Sandakly and colleagues described abdominal lymphadenopathy initially investigated as lymphoma, followed by a diagnosis of brucellosis and radiological improvement (13). Mirijello and colleagues emphasised that lymphoma, brucellosis, and tuberculosis share clinical and imaging features and recommended a multidisciplinary approach to abdominal lymphadenopathy (6). Our patient differed from these reports in three respects: nodal involvement extended above the diaphragm to the supraclavicular and mediastinal regions, the illness was accompanied by a hyperinflammatory syndrome, and nodal regression after treatment was reported by the patient rather than confirmed by imaging.

### 3.2 Comparison with Brucella-associated HLH

Reported cases of Brucella-associated HLH are few and heterogeneous. Fever is the most consistent feature and is usually accompanied by cytopenias and splenomegaly, whereas a clear epidemiological exposure is not always elicited (16). Bone-marrow haemophagocytosis was documented in several reports (14–16). Treatment has ranged from antibacterial and supportive therapy alone to antimicrobials combined with glucocorticoids and intravenous immunoglobulin (16). A 2025 review reported favourable outcomes in the small number of cases identified, although the sample size and likely publication bias preclude reliable estimates of prognosis (17). Relapse after premature discontinuation of anti-Brucella treatment has also been described (16).

The present case departs from this pattern in ways that delayed recognition. Anorexia rather than fever was the presenting complaint, the patient was afebrile on admission, and imaging-defined lymphadenopathy dominated the initial assessment. Without bone-marrow examination, the HLH assessment relied on the HLH-2004 criteria, the HScore, the culture result, and the clinical course. The exposure was identified only after the organism was known. Brucella-associated HLH should therefore be considered even when fever is not the presenting feature and no livestock contact is recorded at admission.

### 3.3 Stepwise diagnostic reasoning and alternative triggers

In adults, the diagnosis of secondary HLH involves two separate questions: whether the syndrome is present, and what has triggered it (7). Our assessment proceeded in three steps (**Figure 4**; **Table 3**). First, infection was established. *B. melitensis* grew in both aerobic bottles from paired sets drawn during fever, and Brucella is not a skin contaminant, so the result indicated true bacteraemia. Second, the syndrome was assessed. Five HLH-2004 criteria and an HScore of 234 supported probable secondary HLH, although three criteria were unassessed and the score was reconstructed retrospectively. Third, alternative or additional triggers were considered.

**Figure 4:**
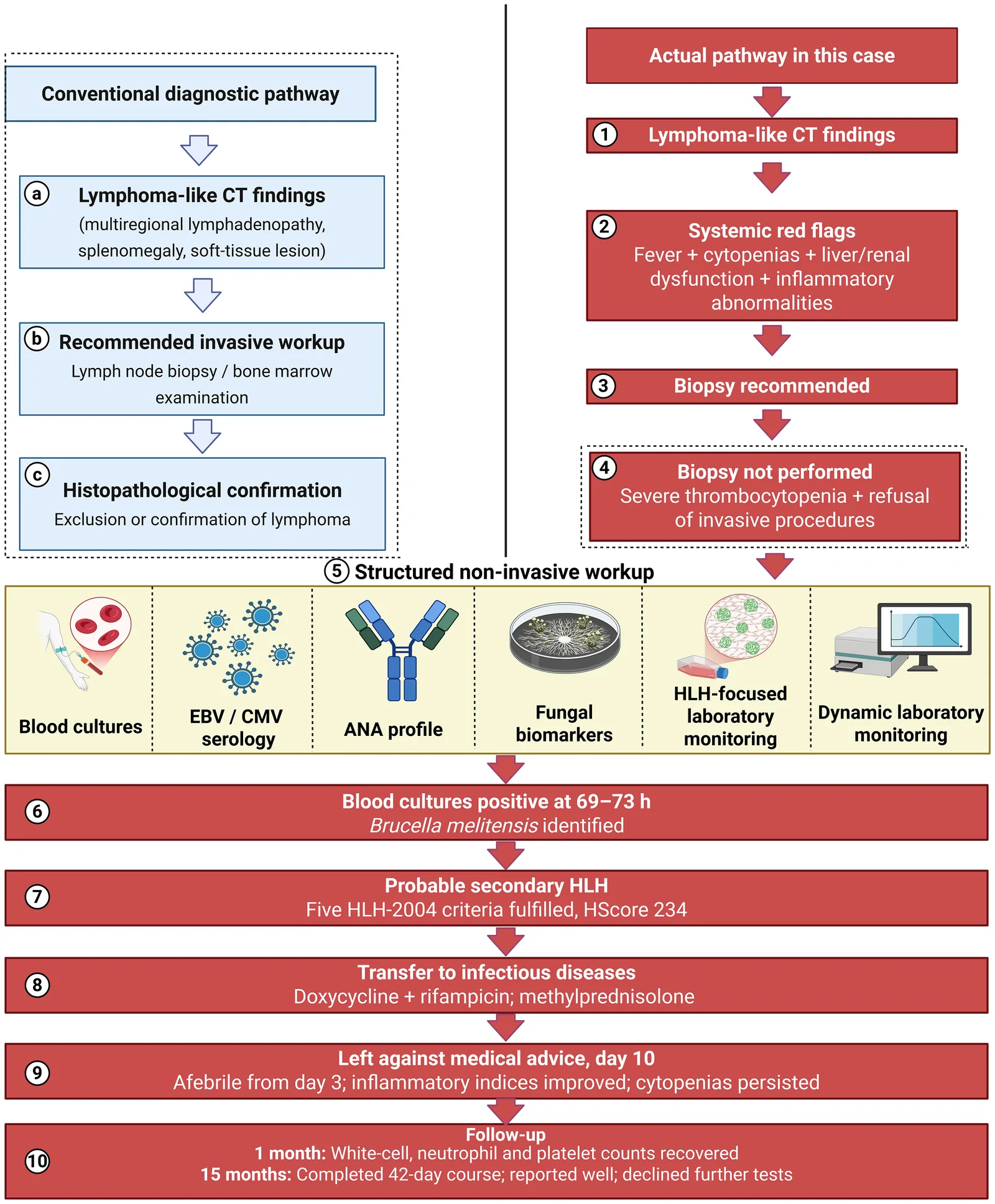
Diagnostic assessment and clinical course. The tissue-assessment and clinical pathways are shown alongside the laboratory investigations. Blood cultures were collected on day 2; *B. melitensis* was reported and directed treatment started on day 6. Day 7 results supported probable secondary HLH; the HScore was retrospective. Follow-up combines blood tests performed at a nearby hospital after the patient returned home, approximately 1 month after discharge, with the patient-reported outcome at approximately 15 months. Abbreviations: ANA, antinuclear antibody; CMV, cytomegalovirus; CT, computed tomography; EBV, Epstein–Barr virus; HLH, haemophagocytic lymphohistiocytosis.

Lymphoma was the most important alternative, because it is the leading malignant trigger of HLH in adults and the CT findings favoured a lymphoid neoplasm (7). Early improvement in inflammatory markers does not exclude lymphoma, because corticosteroids can transiently suppress both lymphoma-related symptoms and lymphoma-associated HLH. Subsequent recovery of white-cell, neutrophil, and platelet counts and the patient’s report of being well at approximately 15 months support clinical improvement. However, corticosteroids were also administered at the subsequent hospital, and their total duration was not established. Neither the laboratory response nor the later symptom report excludes coexisting lymphoma; no repeat imaging or histological assessment was available to establish the nature or resolution of the nodal and peripancreatic abnormalities.

Disseminated tuberculosis was considered less likely on clinical and radiological grounds. The patient reported no previous tuberculosis, and the pre-admission chest CT showed bronchiolitis, minimal fibrotic change, a right lower-lobe bulla, and multiple pulmonary nodules without cavitation or miliary change (**Supplementary Figure S1**). No tuberculosis-specific or tissue investigation was performed. Rifampicin, administered as part of the anti-Brucella regimen, also has antimycobacterial activity, so the later clinical course cannot be taken as evidence against tuberculosis, which therefore remained incompletely assessed.

The autoimmune antibody profile, Epstein–Barr virus and cytomegalovirus IgM, haemorrhagic fever with renal syndrome IgM, and fungal markers were unrevealing for the conditions tested. SFTS remained untested because specimen collection was refused, and HIV testing was not performed because no risk factor was identified in the recorded history. Brucella was therefore considered the leading trigger because the infection was microbiologically proven, it coincided with the hyperinflammatory syndrome, and no positive evidence of another trigger was found. Because some alternatives remained incompletely assessed and malignancy was not excluded histologically, the diagnosis is appropriately described as probable secondary HLH.

### 3.4 Therapeutic rationale

Dexamethasone was first administered on the evening of day 1. Methylprednisolone was started on day 2 during acute deterioration with fever of 41 °C, drowsiness, cytopenias, and hepatic and renal dysfunction, whilst the cause was still under investigation. The progress note records methylprednisolone as an anti-inflammatory adjunct to antibiotics and fluids. The prescription preceded the cytokine and procalcitonin results and the HLH assessment, and was therefore an empirical response to severe systemic inflammation rather than treatment of an established diagnosis. This timing carried two costs. Blood cultures were drawn after antibiotics and corticosteroids had been administered, and early corticosteroids can reduce the diagnostic yield of subsequent node or marrow histology when lymphoma is suspected.

After *B. melitensis* was identified, doxycycline and rifampicin were started to treat the trigger. Methylprednisolone was continued because the cytopenias and the laboratory findings supporting HLH persisted. Adult HLH recommendations advise adapting treatment to the trigger, disease activity, and organ involvement, and note that HLH associated with some intracellular infections responds to specific antimicrobial treatment without an HLH-94 regimen (7). Etoposide was not used, consistent with an infection-driven syndrome and severe thrombocytopenia. Methylprednisolone 40 mg IV daily was continued after discharge. The final taper or cessation date was unavailable.

Doxycycline plus rifampicin is an established oral regimen for brucellosis. A meta-analysis of randomised trials showed that treatment for six weeks or longer reduced failure compared with shorter courses, although doxycycline–rifampicin carried a higher relapse risk than doxycycline–streptomycin (18). The discharge instructions specified a 42-day course and advised continued doxycycline–rifampicin treatment. Continuation and completion were subsequently confirmed, but the exact completion date was unavailable. He also received ceftriaxone and amikacin during continued treatment after returning home. The contribution of each intervention to recovery cannot be separated. Fever and some laboratory abnormalities improved before directed treatment began, during empirical treatment that included corticosteroids and, from day 3, levofloxacin, a fluoroquinolone with *in vitro* activity against Brucella. The platelet measurements spanned a transfusion, and adjunctive herbal treatment was also administered (**Supplementary Table S1**).

### 3.5 Practical implications

The culture intervals have direct practical relevance. Both aerobic bottles became positive only after 69–73 h, and delays of approximately 88 h and 114 h have been reported in Brucella-associated HLH (17). A negative culture at 48 h therefore provides little reassurance when brucellosis remains plausible. Automated systems usually detect Brucella within a routine 5–7-day incubation, but suspected cases should be communicated to the microbiology laboratory because Brucella is a recognised cause of laboratory-acquired infection (5).

Ferritin was first measured on day 7, although cytopenias, splenomegaly, raised LDH, and low fibrinogen were present earlier. This combination should prompt consideration of HLH and an active search for infectious, malignant, and autoimmune triggers (7). Ferritin is inexpensive and widely available, and earlier testing might have broadened the differential diagnosis before biopsy became impracticable. Other laboratory findings required cautious interpretation. Procalcitonin was only modestly raised despite bacteraemia, so a low value did not exclude intracellular bacterial infection. In a paediatric study, HLH was characterised by markedly raised interferon-γ and IL-10 with a moderate IL-6 increase, whereas bacterial sepsis showed IL-6 predominance (19). The IL-6-predominant profile in our patient was compatible with infection-triggered hyperinflammation but was not a specific marker of HLH.

The exposure history also has practical implications. The admission record listed farming, and the relevant exposure setting emerged only when additional questioning identified seasonal street cleaning on a route past sheep farms. Asking about seasonal jobs and the places where patients work, not only their main occupation or direct animal contact, may reveal exposures in endemic regions.

Finally, financial hardship and lack of insurance shaped the investigation, the length of stay, and the follow-up. The patient declined biopsy partly because of cost and left the reporting hospital early, but continued care locally and underwent laboratory follow-up in June and July 2025. He declined further testing at the approximately 15-month telephone follow-up. In similar settings, early discussion of costs and of lower-cost options for follow-up, such as a local blood count and ultrasound, may help preserve objective reassessment.

### 3.6 Strengths and limitations

The strengths of this report are the records-verified chronology, serial laboratory data, transparent reporting of declined procedures and incomplete tests, and follow-up information. The limitations are substantial. Lymphoma and other malignancy were not excluded histologically. Marrow haemophagocytosis, natural killer cell activity, and soluble CD25 were unassessed, and the HScore was reconstructed retrospectively. Brucella serology and nucleic acid testing were not performed, and tuberculosis, SFTS, and HIV were not specifically tested. Anaemia persisted at the last blood test in July 2025, and no repeat imaging established resolution of the nodal and peripancreatic abnormalities. The approximately 15-month outcome remained patient-reported. The durations of subsequent intravenous treatment and the final corticosteroid taper or cessation date were unavailable, and the exposure source remained unconfirmed.

### 3.7 Patient perspective

The patient explained that the cost of biopsy and fear of further physical harm led him to decline the procedure. His household income was very low and he had no medical insurance. These financial pressures also contributed to his and his family’s request for early discharge, despite the recommendation for continued care. After returning home, he continued treatment and attended a nearby hospital for follow-up examinations.

At telephone follow-up, he felt well and no longer noticed swelling in his neck. Further testing seemed unnecessary to him in the absence of symptoms, and the cost and travel were difficult for his household. He preferred to return to hospital if symptoms developed.

## 4 Conclusion

*B. melitensis* infection complicated by probable secondary HLH can present with lymphoma-like generalised lymphadenopathy, splenomegaly, and cytopenias. When tissue sampling is declined or unsafe, blood cultures drawn during fever and incubated beyond 48 h, early ferritin measurement, and systematic screening for alternative triggers can identify a treatable cause. Brucellosis merits consideration when nodal abnormalities and hyperinflammatory findings occur together in a compatible exposure setting, but a non-invasive diagnosis should state explicitly what remains unexcluded and should be followed by objective reassessment whenever possible.

## Data Availability

The original contributions presented in the study are included in the article/**Supplementary Material**. Further inquiries can be directed to the corresponding authors.
