# Supplementary material for: Brucella melitensis bacteraemia with lymphoma-like generalised lymphadenopathy and probable secondary haemophagocytic lymphohistiocytosis under biopsy constraints: a case report
Source: Front Immunol. 2026 Oct 21;17:1975515. doi: 10.3389/fimmu.2026.1975515 (PMC13730908; doi:10.3389/fimmu.2026.1975515)
Supplement: Supplementary file 1 [file Table1.docx]

**Supplementary material**

# Supplementary Table S1. Baseline history and supportive treatment

| **Domain** | **Available information** |
| --- | --- |
| Prior diagnoses and medicines | Subsequent local-hospital records and clarification confirmed type 2 diabetes treated with unspecified medication; this history had not been accurately captured at the initial admission. A local clinic had recorded glucose 7.75 mmol/L and sinus tachycardia and prescribed glucose-, blood-pressure- and heart-rate-lowering drugs. Drug names and doses were unavailable. |
| Immunosuppression | No pre-admission immunosuppressive treatment was documented. HIV testing was not performed; no risk factor was identified on history. |
| Family history | No hereditary disease reported in parents or siblings. Spouse and children reported healthy. |
| Smoking and alcohol | Approximately 20 cigarettes/day for >40 years. Record states alcohol about 4 liang/day, stopped >1 week before admission. Beverage type and alcohol concentration were not recorded; ethanol intake cannot be calculated. |
| Occupation and exposure | The admission record listed farming as the primary occupation. Subsequent questioning established additional street-cleaning work outside the farming season, on a route passing sheep farms. No direct handling of livestock or birth products and no unpasteurised dairy consumption were reported. |
| Financial circumstances | Very low household income and no medical insurance. Cost and concern about physical harm contributed to biopsy refusal and the request for early discharge. |
| Nutritional assessment | Admission screening score 2. On day 3, the nutrition consultation recorded NRS-2002 5, PG-SGA 16 and severe malnutrition by GLIM assessment. |
| Nutrition and fluids | IV fluids and electrolyte replacement. Compound amino acids (18AA), 250 mL IV daily, from day 3 through discharge. Oral nutritional supplementation was recommended on day 3 but initially declined. |
| Gastric protection | Ranitidine 50 mg IV daily, days 1–6; esomeprazole 40 mg IV daily, days 6–10. |
| Hepatoprotective treatment | Glutathione 1.8 g IV daily during admission; magnesium isoglycyrrhizinate 150 mg IV ordered briefly on day 6, followed by compound glycyrrhizin 60 mL IV daily through discharge. |
| Herbal decoction | An oral daily decoction was ordered. The initial formulation ordered on day 1 and repeated in the day 3 note was divided into two daily administrations: Xuanshen 30 g, Dihuang 15 g, Yanzhimu 10 g, Shengshigao 20 g, Chuanxiong 15 g, Danshen 15 g, Danggui 15 g, Cuyanhusuo 15 g, Cu’ezhu 6 g, Cusanleng 6 g, Shancigu 8 g, Banzhilian 15 g, Tubeimu 15 g, Gancao 10 g, Jinyinhua 10 g and Daqingye 10 g. On day 4, Dihuang and Shengshigao were increased to 25 g each, Cu’ezhu and Cusanleng reduced to 4 g each, Jinyinhua increased to 25 g and Daqingye to 15 g; Banlangen 15 g and Chaozhizi 10 g were added. |
| Other adjunctive treatment | Auricular acupressure, moxibustion and acupoint application were recorded. |
| Treatment changes | Levofloxacin was added on day 3 and meropenem on day 4 during empirical treatment. Levofloxacin was stopped on day 6 before the species report; meropenem was stopped later that morning. Methylprednisolone was reduced to 20 mg daily on day 4 and returned to 40 mg daily on day 5. The available notes do not give a specific reason for each of these changes. |
| Corticosteroids and continued local care | Methylprednisolone 40 mg IV daily was continued after discharge for persistent cytopenias. During continued treatment after returning home, the patient also received ceftriaxone 3 g IV daily and amikacin 0.4 g IV daily. He continued doxycycline–rifampicin and completed the prescribed course. |

Herbal ingredient names reproduce the recorded Chinese medicinal names in pinyin. IV, intravenous; NRS-2002, Nutritional Risk Screening 2002; PG-SGA, Patient-Generated Subjective Global Assessment; GLIM, Global Leadership Initiative on Malnutrition.

# Supplementary Table S2. Retrospective HScore components

| **Component** | **Finding used** | **Points** |
| --- | --- | --- |
| Peak temperature | 41 °C, recorded day 2 | 49 |
| Organomegaly | Splenomegaly on CT; no documented hepatomegaly | 23 |
| Cytopenias | Day 7 WBC 1.62 × 10⁹/L, haemoglobin 90 g/L, platelets 47 × 10⁹/L; all three meet HScore thresholds | 34 |
| Ferritin | Day 7 >2000 µg/L; 2000–6000 band used as minimum supported score | 35 |
| Triglycerides | Day 7 2.74 mmol/L | 44 |
| Fibrinogen | Day 7 1.49 g/L | 30 |
| AST | Day 7 167.1 U/L | 19 |
| Known immunosuppression | None documented; HIV testing was not performed. | 0 |
| Marrow haemophagocytosis | Unassessed; conservatively assigned 0 points. | 0 |
| Total | Retrospective reconstruction; approximate model probability 98–99% | 234 |

HScore and HLH-2004 cytopenia thresholds differ. Most laboratory inputs here are from day 7; peak temperature is from day 2 and imaging from before admission. This is not an admission-time score. If ferritin actually exceeded 6000 µg/L, that component would score 50 and the total would be 249 with other inputs unchanged. The original HScore table gives 98% at 230 and 99% at 240.

# Supplementary Table S3. Serial laboratory results

| **Measurement** | **Unit** | **Day 0** | **Day 2** | **Day 4** | **Day 6** | **Day 7** | **Day 10** |
| --- | --- | --- | --- | --- | --- | --- | --- |
| White blood cells | ×10⁹/L | — | 2.21 | 2.19 | 1.61 | 1.62 | 1.29 |
| Absolute neutrophil count | ×10⁹/L | — | 1.06 | 1.54 | 0.65 | 0.51 | 0.57 |
| Platelets | ×10⁹/L | — | 28 | 9 | 30 | 47 | 38 |
| Haemoglobin | g/L | — | 112 | 87 | 87 | 90 | 83 |
| High-sensitivity C-reactive protein | mg/L | — | 48.68 | 55.44 | 17.78 | 12.31 | 8.66 |
| Procalcitonin | ng/mL | — | 0.966 | — | 0.448 | 0.214 | 0.058 |
| Interleukin-6 | pg/mL | — | 553.65 | — | 1.56 | — | — |
| Ferritin | µg/L | — | — | — | — | >2000 | 1452 |
| Lactate dehydrogenase | U/L | 1093 | — | — | — | 397 | 301 |
| Fibrinogen | g/L | — | 1.62 | 1.96 | — | 1.49 | 2.58 |
| Triglycerides | mmol/L | — | — | — | — | 2.74 | 1.74 |
| Alanine aminotransferase | U/L | — | 72.6 | — | 268.1 | 219.9 | 106.0 |
| Aspartate aminotransferase | U/L | 189.5 | 206.9 | — | 298.7 | 167.1 | 46.1 |
| Creatinine | µmol/L | 201 | 135 | — | 79 | 78 | 79 |
| D-dimer | mg/L | — | 21.23 | 13.84 | — | 16.21 | 5.34 |

Day 0 denotes referring-hospital results. Hospital results are placed on report days. Day 2 blood count and liver/renal values were sampled on day 1; day 4 blood count and coagulation values on day 3; day 6 values on day 5. The specimen giving platelets 30 × 10⁹/L was collected before the day 5 transfusion. Creatinine 79 µmol/L and IL-6 1.56 pg/mL were reported before directed therapy began on day 6. IL-6 measurements used different assays and specimen types. Ferritin >2000 µg/L was above the reporting limit. The day 2 cytokine panel also showed IL-10 56.41 pg/mL and interferon-γ 20.18 pg/mL; IL-2, IL-4 and tumour necrosis factor-α were each <2.5 pg/mL. Baseline red-cell count was 3.21 × 10¹²/L.

**Supplementary Table S4. Laboratory follow-up after discharge from the reporting hospital**

A. Serial blood counts

| **Date (2025)** | **WBC ×10⁹/L** | **ANC ×10⁹/L** | **Hb g/L** | **PLT ×10⁹/L** | **MCV fL** |
| --- | --- | --- | --- | --- | --- |
| 12 June | 1.2 | 0.45 | 83 | 44 | 100.8 |
| 15 June | 1.3 | 0.43 | 78 | 76 | 101.0 |
| 20 June | 2.3 | 0.92 | 85 | 78 | 102.8 |
| 23 June | 3.0 | 1.25 | 81 | 72 | 100.8 |
| 1 July | 4.3 | 2.40 | 102 | 176 | 105.3 |
| 19 July | 3.8 | 1.85 | 111 | 131 | 102.2 |
| Reference range | 3.5–9.5 | 1.8–6.3 | 130–175 | 125–350 | 82–100 |

B. Available inflammatory and biochemical measurements

| **Measurement** | **Unit** | **12 June** | **20 June** | **1 July** | **19 July** |
| --- | --- | --- | --- | --- | --- |
| Ferritin | µg/L | 1293 | — | — | — |
| hs-CRP | mg/L | — | 3.18 | — | — |
| ESR | mm/h | — | 28 | 38 | 16 |
| ALT | U/L | — | 11 | 13 | 17 |
| AST | U/L | — | 14 | 16 | 17 |
| Albumin | g/L | — | 29.8 | 40.2 | 39.8 |
| Creatinine | µmol/L | — | 73 | — | — |

The patient underwent these follow-up blood tests at a nearby hospital after returning home. Dates are specimen-collection dates; the reports were issued on the same dates. “—” indicates that no result was available in the supplied records, not a normal or negative result. Blood-count reference ranges are those printed by that laboratory. White cells, neutrophils and platelets were within range on both July dates, but anaemia and increased MCV persisted. Ferritin remained elevated on 12 June; later ferritin results were unavailable. Albumin on 19 July was slightly below the laboratory lower limit of 40 g/L. No repeat imaging was available.

ANC, absolute neutrophil count; Hb, haemoglobin; MCV, mean corpuscular volume; PLT, platelets; WBC, white blood cells; ALT, alanine aminotransferase; AST, aspartate aminotransferase; ESR, erythrocyte sedimentation rate; hs-CRP, high-sensitivity C-reactive protein.

# Supplementary Figure S1. Chest CT in lung windows.

(A–D) Representative axial chest CT images at progressively inferior thoracic levels. These images supplement the pre-admission CT findings described in Section 3.3.
